# Diphtheria and Bacteria

**Published:** 1881-06

**Authors:** P. P. Wells

**Affiliations:** Brooklyn, New York


### DIPHTHERIA AND BACTERIA.

P. P. WELLS, M.D., BROOKLYN, NEW YORK.

HYPOTHESIS has been a great barrier in the way of medical progress, if not the greatest with which it has had to contend in all its history. It has always been easier to imagine how a matter may be, or may have been, than to find out how it is or was by careful collation and examination of facts, and from these construct a whole of truth based on this only sure foundation. And then it would seem to be so easy, having wrought out the imagination into a seeming of reality, to mistake it for this, and to proceed to reason and act upon it as if there could be no mistake about it. And further, that a protracted contemplation of the figment so blinds the mind of the inventor that all who do not readily accept his alleged discoveries are, by him, consigned to the category of the hopelessly stupid, or incorrigibly obstinate. The figment becomes, perhaps on the principle of love of offspring, a fact to its author, and the essential difference between fact and fancy becomes to him, so far as his pet is concerned, a matter beyond his grasp. It is notorious, that medical history has come down to us composed almost wholly of a succession of theories, each giving place to a successor, to be referred to the limbo of the false and useless, and so on, till now that vast receptacle well-nigh contains them all. And after all this, continued through all these centuries, each hypothesis giving place to another equally as false as itself, the medical world has not lost its love of theory. It has not learned, as it should, and might, long ago, that fact, and fact alone, is that which can bring to us increase of knowledge of the least practical value.

These thoughts have been suggested by reading a pamphlet of a few pages entitled, "Diphtheria and Bacteria," by Rollin R. Gregg, M.D., of Buffalo, N. Y., which seems to have been born so largely of the imagination of its author as to serve well for the occasion of a protest against a continuance of the history of the old school, in this particular, in the life and literature of the new. It has been our fortune to be present in many medical organizations where diphtheria has been discussed by many doctors, and on leaving each, the one reflection has come—how little these discussions have added to one's positive knowledge of the disease or its cure. This has been true of each one of them. This was a matter of course when the whole, or nearly the whole, was made up by each doctor telling what he had done in treating cases which had been subjects of his care. There was a certain interest in this. It is well enough to know what one's neighbors have been doing. But in all these discussions it is not now remembered that any one of these many doctors told *why* he did what he did. I have no recollection of any one showing that the agents he employed in treating his cases were, in their action on the living organism, more like the phenomena of his cases than any others, or that they were like at all. Yet these organizations were all called *homœopathic*. They had given this, that and the other, because they had read, or had been told it would cure this troublesome and dangerous plague; yet they were made to know in the sequel that it did not cure. The difference between them and the writer of the pamphlet before us is considerable. These told what they *did* with and for the disease. Dr. G. tells us what the disease is: or, at least, he tells us what he thinks it is: and in doing this he has so fallen into the sin of the old school of giving fancy for fact, that, but that we know his belongings, we should not think of questioning his orthodoxy as judged by the leading ones of that school. Equal to any of them, even the oldest, he tells what *is*, as though it were a fact he had proved, leaving his readers in ignorance of the steps which have brought him to his conclusions. If the reader accepts this it must be on the authority of the writer, and not because of any facts which accompany it.

Now, whatever of ingenuity may be conceded to Dr. G., and we would grant him much, we are constrained to think it not unreasonable to demand something in the way of proof of the soundness of his theorizing—for this is what his pamphlet is—before it can be accepted as adding to the sum of our knowledge of this most interesting and important subject.

This is what the doctor says: "In the first place, then....

the fact should be born distinctly in mind that the characteristic exudations of diphtheria....are wholly, or principally, fibrin, Oertel, Virchow, and all other prominent writers upon the subject assert such to be the case." Then having given the mode of action of fibrin in forming clot, he proceeds to assert, that "fibrin is always in excess in the blood in diphtheria." How does he know this? So far as the pamphlet goes, this stands wholly on the *ipse dixit* of Dr. G. This may be just as he says, but he has given no proof. He declares further, that this excess is a fact in "all other inflammatory diseases;" that excess of fibrin is "always a source of more or less danger.... in pleuritis, peritonitis, etc." How does he know this? Is it not reasonable to suppose that the danger in inflammation of these serous tissues, is as much from the fact, that whereas in their normal state they separate from the blood only so much of its serum as is needed in the discharge of their functions, now, in the changed action of these membranes, in their diseased condition, they eliminate another element, fibrin, as to charge this increase of danger to *excess of this element in the blood?* If fibrin be in excess, for which we have only the doctor's word, he has failed to connect danger with this fact, except so far as he has attempted to make it responsible for embolism, which may give much trouble, no doubt, if present, and the doctor says that in consequence of this "a goodly number of diphtheritic patients perish." This may be so; but it is certainly true that we have never met with *one.* The doctor gives us no signs by which he has decided that death has been adjudged by him to have resulted from this cause, and therefore we are left wholly without the means of judging as to the accuracy of his decision. Neither in a rather long practice have we known inflammation of the pleura or peritoneum to terminate fatally from this cause, which is certainly a little remarkable, if these obstructions are a result of this excess, and this excess is present in *every* case of these diseases as well as in diphtheria. We have no recollection of any fatal case of either of these diseases, which any amount of ingenuity, aided by the notion of this excess and its possible consequent embolism, could have dragged into the category of deaths by thrombosis.

This being the fact, it would be difficult to avoid the conclusion, either that the alleged excess is much less common than the doctor represents, or that it is much less important than he supposes. Having declared the invariable presence of excess of fibrin in every case of diphtheria, and having told how this excess in the circulation is disposed to roll itself into clots, and how these clots by becoming plugs in the larger or smaller arteries, are a source of great danger to the patient (he would seem to regard it as a principal one), the doctor has prepared the way for his grand idea, that indeed, for which it would seem his paper was written. The patient having to struggle with this excess, and its tendency to roll itself into clots, "*Nature*," being "*conservative and preservative of life under all circumstances, where she possibly can be. Hence, as fibrin is always in excess in the blood in diphtheria, and as all heart clots and all false membranes of the disease are of fibrin, and the former, or heart clots, are so extremely dangerous, there can be no question that the exudation and formation of all the membranes of the disease are the result of nature, or the preservative forces of life, stepping in and expelling the excess of fibrin from the blood to prevent its accumulation in the circulation to such an extent as to form coagula there that are so fatal.*"

There it is—put in plain English it amounts to just this: the awful membrane of diphtheria, which has been and is the terror of so many worthy doctors, is wholly a beneficent arrangement of nature to protect the heart and arteries from embolism! It is well that this is known at last, for if true it may save some of us, who did not know it before, from painful fears and anxieties hereafter—those of us, we mean, who have been accustomed to estimate the danger of our cases as in some degree disclosed by the greater or less extent of the exuded membrane. This is all wrong! It is only a beneficent arrangement of nature to get rid of excess of fibrin in the blood-vessels, and this in the interest of heart protection from thrombosis!

Hereafter we may know that this object of our great fears is only a "conservative" resort of nature, and "not a lion at all, ladies and gentleman, but only Snug, the joiner." If you ask, how are we to know this? we can only reply, Dr. G. has said it, and we know no other reason for believing it. We have said, if this grand idea of Dr. G. be true, we may dismiss our fears of the membrane hereafter. But is it true? Is the exudation of this, our great dread, beneficent, and not a danger after all? If this be so then one of the most obvious evidences of this beneficent intent and tendency, and one for which we should first look, and most readily recognize, would be a relief to the patient, beginning with the deposit, and proportioned, always, to the extent of this. Has any man seen such relief, so beginning and so progressing, becoming greater and greater as the membrane was more and more extended? Has not the experience been rather, that the danger has been in proportion to the extent of the exudation, than that this by increase has brought relief in any degree? The idea that "nature," here and so, steps in, with beneficent intent, is certainly an interesting one, but its practical value is materially diminished by the fact that this most excellent lady sometimes "steps in" too far. She, seemingly, does not know when to stop. She "means well, but don't know." This is evidently true of those cases where the exudation extends to the respiratory tubes and air cells. The certain fatality of cases where the pulmonary tissue is so invaded, would seem to intimate that "nature" did not know where to put this alleged excess, which we have been told is so important a factor in the item of danger in this formidable disease. In filling the air cells with this excess, she certainly made a bad use of it, if, indeed, she did not make a consummate blunder. Then, the doctor gives, as instances of the danger from excess of fibrin in the blood, pleuritis and peritonitis. Does he also claim that the deposit of fibrin in these diseases, on the serous surfaces involved, is a beneficent fact? Does he not know that the danger in these diseases is generally thought to be enhanced by the increase of the deposit, and to be in no small degree determined by the extent of this; *i. e.*, the greater the exudation the greater the danger? Here, again, "nature" often "steps in" too far, or, rather, here the doctor's theory of her beneficent action fails as completely, as an intelligent observation of the facts will show that it does in diphtheria. This is a pity, for if true, it might save many of us many a heart-ache. Again, having shown how benevolent and beneficent the deposit of this exudation is, which so pleasantly and safely relieves us of our apprehension from excess of fibrin in the blood, he proceeds to assure us that were not this excess "expelled from the circulation, *every*" (the italics are the author's) "case of diphtheria would prove fatal," etc., and this "in a few days," and by reason of "the formation of large coagula in the heart, . . . . or small ones that would lead to incurable inflammations, and cause death in that way." He also says that death is prevented in pleuritis and peritonitis by this same resort, *i. e.*, by the exudation of the fibrin on these serous surfaces.

The first remark we have to make on this assurance as to diphtheria, is, we do not believe it, and for this reason: there are cases met in most epidemics of this disease, showing all its symptoms except the one of exudation, and followed sometimes with its troublesome sequelæ, notably by paralysis; and though there has been no deposit of membrane, the patients have not died. More than this, there has been no apparent increase of danger from the absence of the usual exudation; therefore we do not believe this assertion. We do not believe it because we know it is not true in these cases. We do not believe it in the case of serous inflammations, because in a practical experience of more than forty-seven years, in which we have treated our share of such cases, we can not recall one fact which in the least confirms the truth of it. The doctor invokes a treatment of this disease "more in accordance with these incontrovertible facts than it has been in the past." If by this be meant a treatment which shall increase the extent and amount of the membrane—and we can not see what else he can mean—we can only say in reply, God forbid! The doctor gives us no hint as to what this treatment shall be, nor what the means he would have employed to accomplish this great disaster.

Having disposed of *all* such cases of these inflammations as are not relieved of this excess of fibrin, by exudation, or otherwise, and satisfactorily given them over to a necessarily fatal termination, he tells of bacteria; at least he tells what he thinks of what others have found by the use of the microscope, in connection with this and some other diseases, and which they have called bacteria. Dr. G. has found out, or he is mistaken, that these heretofore supposed little living organisms are only *fibrin!* He assures us "there is not the slightest proof to show that these bodies are vegetable parasites, or organisms." We never attached much importance to the presence of these bodies in the secretions or on the surfaces of the sick, and none at all to them as a cause of which diphtheria was the effect. But when the doctor says "there is not the slightest proof," etc., he is certainly mistaken. There has been, and is, very substantial and respectable testimony to the veritable existence of these organisms in diphtheria. When their presence and importance were discussed in the World's Homœopathic Convention, in 1876, I remember a representative to that convention from Germany, a professor in an institution in his own country, his duties as teacher compelling his use of the microscope, and familiarity with the objects of its revelations, declared that he had seen them, *and knew what he saw.* He was a man of remarkable acuteness and clear in his statement of facts, and I have no doubt truthful. It is more than likely he was quite familiar with the modes and actions of fibrin while gathering into a clot, as it is some time since this ceased to be a novelty. And yet he says of bacteria, "I have seen them and I know." The case being so, we are compelled to believe in the little organisms, though Dr. G. knows of no proof of their existence. We have never seen them, but the Herr Professor saw them and was abundantly competent to testify as to what he saw. There are two assertions in this pamphlet, resting, so far as it is concerned, wholly on the *ipse dixit* of Dr. G., and these contain all there is in it which is new: 1st, that there is an excess of fibrin in the blood in all cases of diphtheria; 2d, that the exudation of this in the form of membrane, in diphtheria, is a beneficent fact, because, but for this there would certainly be cardiac thrombosis in every case of the disease, with certain death as the result. It is a sufficient remark on this first asertion—for, so far as this pamphlet is concerned, this is all there is of it—that if the fact be admitted of this universal excess, for the sake of the argument, this is not the only, nor the most importent factor the prescriber has to meet and deal with in solving the problem of the simillimum for his case. The tendency to a rapid dissolution of both solids and fluids in the severer forms of the disease, is a fact before which this alleged excess becomes of very small importance. The second assertion, that of the beneficent character of the membrane in diphtheria has had from us all the consideration it merits, if not more. We will only add our belief that the judgment of the best practical minds of our school will fully confirm our own conviction, that this is but a figment of Dr. G.'s imagination, pure and simple—that it is just this and nothing more. If the above are just judgments as to the value of these assertions, then the question arises immediately as to their *raisons d'etre*—why in print? We can see no other or better answer to this than the supposition that the doctor really thought he had made a great discovery in this beneficent imagination, and that pride of paternity so completely blinded him as to its true character as to render him wholly insensible to the difference between fact and fiction. In the pamphlet before us its reader is referred for a knowledge "of the cause of fibrin being brought into excess in the blood" ....and of "the question of treatment....to my late published work on the subject." So it appears that this hypothesis and its no less hypothetical consequences were intentionally left resting on the author's sole and unsustained *dictum*. Practically it was as well to leave it so as any other way. Of treatment he says nothing here, except to acknowledge it "the more practical part of the subject." This is not only true, but more than this, it is the only practical part of the subject till it has been shown how the hypotheses of this pamphlet can be made to affect favorably the treatment of this formidable disease. If the object of these eight pages was to call attention to "my late published work on this subject," with a view to enhancing its sale, then its motive is clear enough; and hypothesis and treatment being by it left where they are, it is difficult to conceive for it any other. Now, as to the homœopathic treatment of diphtheria.

We have said, in the many discussions of this we have heard in homœopathic bodies by homœopathic physicians, they have been almost exclusively made up of statements of what individuals had done for the cure of the cases they had to care for. It is not a little remarkable, when we remember all these physicians professed to be guided and controlled in their prescriptions by our law of therapeutics, that hardly any two of them had done the same thing, and that few of them seemed to have had a better foundation for what they had done than this: they had been told, or had read, that this, that, or the other was *the* cure for diphtheria, and they made haste to give it. We say this is not a little remarkable, if we remember they all had the same law for their guide, if they would but give heed to it, and that this they believe, or profess to believe, is equal to all their needs in other cases; yet in this of diphtheria, each has made greater haste than the other to cast the law behind his back, though, just here they have needed its guidance more, far more, than elsewhere. It is as though before this fatal malady they had been so seized with panic as to forget the universality of the law of cure they had professed faith in, and had been ready to do any thing recommended by any body, rather than to proceed according to the requirements of the law to analyze their cases, and find for them their required simillimum. Why should this be, unless it be shown first that this disease stands an exception to the universal relationship existing between all other diseases and their curatives, found, and found only, in the similar facts of the diseased and drug action? We submit that this disease is no exception, and shows none in its relationship to law, or in its response to impressions of similar curative agencies. It stands before the law and the prescriber just like any other and every other disease he may be called to treat. Further, that it responds to its curative like any other. This is proved in the history of an epidemic of uncommon severity which prevailed in a neighboring city a few years ago. The fatal cases under allopathic treatment were more than fifty per cent. of all so treated; while under the average of homœopathic treatment, so called, the loss was but sixteen per cent.; and in the same epidemic three physicians treated over two hundred and forty cases without a single death. When told of this successful practice, the result seemed so extraordinary as to be incredible. Two of these physicians were personal friends of the writer, and the first time he saw one of them, after hearing the remarkable fact (Hering), he asked his friend if this were true. It was a surprise to hear him say it was. He confirmed the statement fully, and added, "these were genuine cases of fully developed diphtheria, treated by us, and does not include the multitude of sore throats which we treated, and which lacked the characteristics of diphtheria." We asked, "how was this? what did you do?" He replied, "We analyzed *every case*, and gave the required similar remedy when we had found it, and left it to do its work."* And here was the whole secret of it. This epidemic prevailed soon after it was proclaimed that the *protoiodide of Mercury* had been found to be *the* specific for this disease: a

* The two physicians who, with the late Dr. Hering, achieved this, we believe, unexampled success, in curing this disease in its malignant epidemic form, were Dr. Reichelm and Dr. Ad. Lippe.

claim for this drug which many have not yet learned is far beyond its merits. It is hardly unreasonable to suppose that as the claim was then *new*, and generally hopefully believed in, that these sixteen per cent. losses all had the drug and then—died—and homœopathy failed in each case. Not so, as appears from this statement of Hering, when asked what remedies they found most frequently called for, he replied, Mercury almost never, in any form, and least of all the protoiodide. Here was how the success came. These were not the men to abandon law and run after a new thing because somebody had said it would cure. They kept to the law, and the result justified them and the law. They ran after no will-o'-the-wisp of a hypothesis, because it happened to be an ingenious one; in this, as well as in their loyalty to law, giving an example worthy of the following of all who love truth more than fiction or novelty; and a promise of success to all who will go and do likewise; which, it is submitted, no other course of proceeding can excel or equal.

That diphtheria stands before the law, when a subject of medical treatment, just like any other disease, and responds to the impress of its simillimum just like other diseases, is shown in the case treated by the writer, the patient being our late honored and loved colleague, Dr. Carroll Dunham. He was attacked at his home in Newburgh, N. Y., and was brought by steamer to Brooklyn, for treatment by the writer. He was found in extreme prostration, with hot skin and rapid pulse; throat red and greatly swollen, with patches of whitish grey exudation on tonsils and fauces; swallowing extremely painful and difficult; drowsiness so great he could not be kept awake more than a few minutes; he fell asleep almost as soon as he had ceased trying to speak, and in three or four minutes he would wake with a sigh and say: "How much better I feel!" and sleep again almost immediately. This *feeling better after sleeping*, was the first symptom we investigated, and found it only in a marked degree credited to a drug we had never heard recommended for diphtheria, and in our own mind had never been associated with it as a possible curative. We did not accept it for this reason, but proceeded to look for the drug which had most of the other symptoms, i.e., those which were peculiar to the case, which I can not now recall, and were greatly surprised to find them one after another, range themselves under this same drug. When this was ascertained there was no longer hesitation in giving it, though I did not know then it had been given in any case of this disease before. As it turned out, this made not the slightest difference in the result. In twelve hours all the most painful symptoms had disappeared, with much of the extreme exhaustion, exudation and fever. There was only some redness and swelling in the throat remaining, for which, after careful examination he got a single dose of another remedy, and this completed the cure considerably inside of forty-eight hours. This severe case, for it was severe, judged by the sufferings of the patient, or by his constitutional manifestations, was perfectly cured in this time with only two doses of medicine, and one of them a supposed stranger to the disease. It teaches the mastery of the simillimum even here; and further, that having given this, there need be no nervous anxiety to repeat doses or to add to it other drugs; and more than this, that no hypothesis, however ingenious, can add to the curing power of the specific—whether this has ever been given to a similar case or not.

